# Assessment of Genetic Variation and Population Structure of Diverse Rice Genotypes Adapted to Lowland and Upland Ecologies in Africa Using SNPs

**DOI:** 10.3389/fpls.2018.00446

**Published:** 2018-04-09

**Authors:** Marie Noelle Ndjiondjop, Kassa Semagn, Mounirou Sow, Baboucarr Manneh, Arnaud C. Gouda, Sèdjro B. Kpeki, Esther Pegalepo, Peterson Wambugu, Moussa Sié, Marilyn L. Warburton

**Affiliations:** ^1^Africa Rice Center (AfricaRice), Bouaké, Côte d’Ivoire; ^2^Department of Agricultural, Food and Nutritional Science, University of Alberta, Edmonton, AB, Canada; ^3^AfricaRice, Ibadan, Nigeria; ^4^AfricaRice, Saint-Louis, Senegal; ^5^Genetic Resources Research Institute, Kenya Agricultural & Livestock Research Organization, Nairobi, Kenya; ^6^AfricaRice, Antananarivo, Madagascar; ^7^Corn Host Plant Resistance Research Unit, United States Department of Agriculture-Agricultural Research Service, Starkville, MS, United States

**Keywords:** ARICA, NERICA, *Oryza glaberrima*, genetic differentiation, rice ecology, SNPs

## Abstract

Using interspecific crosses involving *Oryza glaberrima* Steud. as donor and *O. sativa* L. as recurrent parents, rice breeders at the Africa Rice Center developed several ‘New Rice for Africa (NERICA)’ improved varieties. A smaller number of interspecific and intraspecific varieties have also been released as ‘Advanced Rice for Africa (ARICA)’. The objective of the present study was to investigate the genetic variation, relatedness, and population structure of 330 widely used rice genotypes in Africa using DArTseq-based single nucleotide polymorphisms (SNPs). A sample of 11 ARICAs, 85 NERICAs, 62 *O. sativa* spp. *japonica*, and 172 *O. sativa* spp. indica genotypes were genotyped with 27,560 SNPs using diversity array technology (DArT)-based sequencing (DArTseq) platform. Nearly 66% of the SNPs were polymorphic, of which 15,020 SNPs were mapped to the 12 rice chromosomes. Genetic distance between pairs of genotypes that belong to indica, japonica, ARICA, and NERICA varied from 0.016 to 0.623, from 0.020 to 0.692, from 0.075 to 0.763, and from 0.014 to 0.644, respectively. The proportion of pairs of genotypes with genetic distance > 0.400 was the largest within NERICAs (35.1% of the pairs) followed by ARICAs (18.2%), japonica (17.4%), and indica (5.6%). We found one pair of japonica, 11 pairs of indica, and 35 pairs of NERICA genotypes differing by <2% of the total scored alleles, which was due to 26 pairs of genotypes with identical pedigrees. Cluster analysis, principal component analysis, and the model-based population structure analysis all revealed two distinct groups corresponding to the lowland (primarily indica and lowland NERICAs) and upland (japonica and upland NERICAs) growing ecologies. Most of the interspecific lowland NERICAs formed a sub-group, likely caused by differences in the *O. glaberrima* genome as compared with the indica genotypes. Analysis of molecular variance revealed very great genetic differentiation (*F*_ST_ = 0.688) between the lowland and upland ecologies, and 31.2% of variation attributable to differences within cluster groups. About 8% (1,197 of 15,020) of the 15,020 SNPs were significantly (*P* < 0.05) different between the lowland and upland ecologies and formed contrasting haplotypes that could clearly discriminate lowland from upland genotypes. This is the first study using high density markers that characterized NERICA and ARICA varieties in comparison with indica and japonica varieties widely used in Africa, which could aid rice breeders on parent selection for developing new improved rice germplasm.

## Introduction

In Africa, rice is a staple food for millions of people and constitutes a major part of the diet in the continent (Maclean et al., 2002; Atera et al., 2011). Rice belongs to the genus *Oryza*, which consists of several wild and two cultivated species. *Oryza sativa* L. (Asian rice) and *O. glaberrima* Steud. (African rice) are the two cultivated rice species in Asia and Africa, respectively (Chang, 1976). Several African rice accessions have adaptive or protective mechanisms of resistance/tolerance to major abiotic and biotic stresses, including drought, iron toxicity, weed competitiveness, nematodes, African rice gall midge, and bacterial blight (Jones et al., 1997b; Linares, 2002; Vikal et al., 2007). However, African rice has its own weakness, including lodging, limited number of spikelets per panicle, grain shattering, and prolonged seed dormancy (Ndjiondjop et al., 2012). The Asian rice was probably introduced into West Africa at the beginning of the 16th century and adopted by farmers living in the Upper Guinea Coast who had previous experience in growing the African rice (Linares, 2002). However, most Asian rice varieties have limited resistance/tolerance to locally endemic abiotic and biotic stresses in Africa (Jones et al., 1997a).

To combine traits of economic importance from both Asian and African rice, interspecific breeding programs were initiated by the Africa Rice Center (AfricaRice) breeders, formerly known as the West African Rice Development Association (WARDA), in the early 1990s. Using *O. glaberrima* as donor parents and *O. sativa* as recurrent parents, AfricaRice breeders developed various improved interspecific rice varieties via backcrossing, of which 78 varieties have been nominated and/or released as NERICAs. NERICAs are adapted to upland, irrigated lowland or rainfed lowland ecologies, and combined the high yield potential from Asian parents and resistance to abiotic and biotic stresses from the *O. glaberrima* parents (Jones et al., 1997a). Due to shared parentage among NERICAs, there has been a concern about the extent of genetic differences among the released NERICA varieties (Orr et al., 2008). In two separate studies conducted on 18 upland NERICAs genotyped with 102 microsatellite markers (Semagn et al., 2006) and 48 lowland NERICAs genotyped with 60 microsatellite markers (Ndjiondjop et al., 2008), we previously reported huge molecular variation amongst most pairs of NERICAs. Those molecular studies, however, were conducted either on a small number of NERICAs and/or using a limited number of microsatellite markers.

Through an effort named the Africa-wide Rice Breeding Task Force (ARBTF), AfricaRice established a multi-location rice testing network spanning 30 countries in 2010 whose objective was identifying and releasing superior rice varieties with consumer-preferred traits. Those released through the ARBTF network are called ARICAs (Advanced RICe for Africa). Through the above effort, several varieties were released, including 18 ARICA varieties developed from intraspecific crosses between Asian rice parents (16 ARICAs) and interspecific crosses involving *O. glaberrima* and *O. sativa* parents (ARICA 4 and ARICA 18^1^). However, the ARICAs have not been characterized using modern high density molecular markers. Hence, there is a need to understand the extent of molecular variation amongst NERICA and ARICA varieties, and how they differ from other *O. sativa* varieties/accessions that are widely used in Africa.

Genetic diversity, relationship and population structure studies are useful for different purposes, including the selection of parental combinations for creating progenies that are phenotypically superior and with significantly higher yield potential compared to their parents (Barrett and Kidwell, 1998; Mohammadi and Prasanna, 2003). Single nucleotide polymorphism (SNP) markers have become popular for molecular characterization of various species. SNP data can be obtained using uniplex assays, multiplex assays, and genotyping by sequencing (GBS) methods based on next generation sequencing technology (Semagn et al., 2014, 2015). The diversity array technology (DArT)-based sequencing (DArTseq) (Sansaloni et al., 2011) is a GBS method for genotyping individuals with high density SNPs, which has been used in rice (Courtois et al., 2013; Ndjiondjop et al., 2017), maize (Chen et al., 2016; dos Santos et al., 2016), durum wheat (Baloch et al., 2017), rye (Al-Beyroutiová et al., 2016), water melon (Yang et al., 2016), and pineapple (Kilian et al., 2016). Recently, we studied the genetic variation and population structure of 2,179 *O. glaberrima* accessions conserved at the AfricaRice genebank using 27,560 DArTseq-based SNPs. We found out that (i) only 14% (3,834 of 27,560) of the SNPs were polymorphic across the whole *O. glaberrima* collection, which is much lower than diversity reported in other *Oryza* species; and (ii) a subset of 350 accessions selected to represent a mini-core collection captured 97% of the SNP polymorphism and nearly all alleles observed in the whole *O. glaberrima* collection available at the AfricaRice genebank (Ndjiondjop et al., 2017). The objective of the present study was to investigate the genetic variation and population structure of 330 genotypes representing NERICA, ARICA, *O. sativa* spp. *indica*, and *O. sativa* spp. *japonica* that are widely used in Africa using SNP markers.

## Materials and Methods

A total of 330 diverse rice varieties and accessions (here referred to as genotypes) widely used in sub Saharan Africa were used in the present study (Supplementary Table S1). The germplasm used in the present study includes 234 *O. sativa* genotypes (172 indica, and 62 japonica) that are either widely grown by farmers in sub Saharan Africa, and/or extensively used as trait donors by rice breeders in the region; and 96 genotypes developed by AfricaRice breeders since the 1990s. The genotypes developed by AfricaRice includes 85 NERICAs derived from interspecific crosses involving *O. sativa* and *O. glaberrima*, 2 ARICAs (ARICA4 and ARICA18) developed from interspecific crosses between *O. sativa* and *O. glaberrima*, and 9 ARICAs developed from intraspecific crosses involving *O. sativa* parents. As shown in Supplementary Table S1, the 330 genotypes are adapted to different rice ecologies in Africa, which includes the upland (81), irrigated lowland (89), rainfed lowland (161), and mangrove lowland (1) growing environments. Overall, 1 ARICA and 19 NERICA with japonica parents plus all japonica genotypes are grown in the upland ecology, while 10 ARICA and 66 NERICA with indica parents, and all indica genotypes are grown in the lowland (rainfed, irrigated or mangrove) ecologies.

Genomic DNA was extracted from a single 3-weeks old seedling using the cetyltrimethyl ammonium bromide (CTAB) method (Murray and Thompson, 1980), with minor modifications as recommended by DArT^2^. DNA samples were genotyped at the DArT P/L laboratory using DArTseq^TM^ technology (Sansaloni et al., 2011; Ren et al., 2015) as described in our previous paper (Ndjiondjop et al., 2017). We received imputed data of 27,560 SNPs (summarized in **Table 1**) from DArT Pty Ltd., which were polymorphic across 5 *Oryza* species sampled from the AfricaRice gene bank. We filtered SNPs using a minimum minor allele frequency (MAF) of 0.01 across the 330 genotypes in TASSEL v.5.2.37 software (Bradbury et al., 2007). There were 18,186 polymorphic SNPs, of which 82.6% (15,020 SNPs) mapped to the 12 rice chromosomes and the remaining 17.4% (3,166 SNPs) were not assigned onto any of the chromosomes (**Table 1**). Genetic distance matrices between pairs of the 330 genotypes were calculated with all 18,186 SNPs and the 15,020 mapped SNPs using the identity-by-state (IBS) method implemented in TASSEL v.5.2.37 (Bradbury et al., 2007), and Mantel correlation was calculated between the two distance matrices using XLSTAT 2012 (Addinsoft, New York, United States^3^). As correlation between the two distance matrices was 0.99, we retained the 15,020 mapped SNPs for all subsequent statistical analyses (Supplementary Table S2). To understand the molecular variation and relationships among genotypes that belong to different categorical groups (e.g., types of rice, ecology, predicted groups from cluster and population structure analyses), we also created several sub-input files from the 15,020 SNPs that were polymorphic among genotypes belonging to the same category. All SNP with a minor allele frequency of ≥0.01 were considered polymorphic.

**Table 1:** Summary of the single nucleotide polymorphism (SNPs) used for genotyping and the polymorphic SNPs used for statistical analyses.

Chromosome	Initial number of SNPs used for genotyping	Number of polymorphic SNPs	Number of polymorphic SNPs across 330 genotypes	Number of polymorphic SNPs within indica	Number of polymorphic SNPs within japonica	Number of polymorphic SNPs within NERICA	Number of polymorphic SNPs within ARICA	Physical length based on 27,560 SNPs (kb)	Map length per marker (kb)
1	2,731	1,679	1,679	1,554	1,422	1,506	1,224	43,230	26
2	2,446	1,495	1,495	1,395	1,223	1,350	1,059	35,885	24
3	2,318	1,546	1,546	1,324	1,174	1,402	1,069	36,413	24
4	2,062	1,353	1,353	1,180	1,005	1,228	878	35,498	26
5	1,681	946	946	884	807	834	699	29,763	31
6	1,866	1,282	1,282	1,139	919	1,182	777	31,191	24
7	1,780	1,045	1,045	960	906	929	751	29,679	28
8	1,654	1,432	1,432	1,058	1,032	1,316	832	28,429	20
9	1,375	857	857	816	712	776	707	22,947	27
10	1,444	894	894	726	726	805	604	23,205	26
11	1,839	1,432	1,432	1,162	1,038	1,330	928	29,000	20
12	1,719	1,059	1,059	1,007	844	943	747	27,505	26
Unmapped	4,645	3,166	–	–	–	–	–	–	–
Total	27,560	18,186	15,020	13,205	11,808	13,601	10,275	372,746	


Genetic purity, relative kinship, cluster analysis, principal component analysis, the model-based population structure analysis, analysis of molecular variance, and maximum length sub-tree were conducted as described in our previous study (Ndjiondjop et al., 2017). In addition, we used the hierarchical island model implemented in ARLEQUIN v.3.5.2.2 to detect loci that may have undergone selection (Excoffier et al., 1992). The island model has been reported to be robust to uncertainties about the exact number of groups in the data (Excoffier et al., 2009).

## Results

### Marker Polymorphism and Genetic Purity

The number of mapped SNPs that were polymorphic across 330 genotypes varied from 857 on chromosome 9 to 1,679 on chromosome 1 (**Table 1**), with an average of 1,252 per chromosome. Average minor allele frequency was 0.192. The physical length of each chromosome varied from 22,947 kb on chromosome 9 to 43,230 kb on chromosome 1 (Supplementary Figure S1), with a total genome size of 373 Mb. As shown in **Table 1**, the average map distance among adjacent markers (inter-marker interval) varied from 20 to 31 kb, (i.e., there was at least one polymorphic SNP within every 20–31 kb physical interval). The number of polymorphic SNPs within each type of rice out of a total of 15,020 was 13,205 for indica, 11,808 for japonica, 13,601 for NERICA and 10,275 for ARICA (**Table 1**). Residual heterozygosity per genotype varied from 0.6 to 20.1%, with an overall average of 1.4% (Supplementary Table S1). Approximately 95% of the genotypes were considered genetically pure with <3.2% residual heterozygosity, which is the expected average residual heterozygosity for lines extracted from S_5_ or later generations. About 4% (13 genotypes) had residual heterozygosity ranging from 5.5 to 20.1%, which may indicate the need for further selfing/purifying and the possibility of error during seed handling or increase in these genotypes.

### Genetic Distance and Relatedness

**FIGURE 1:**
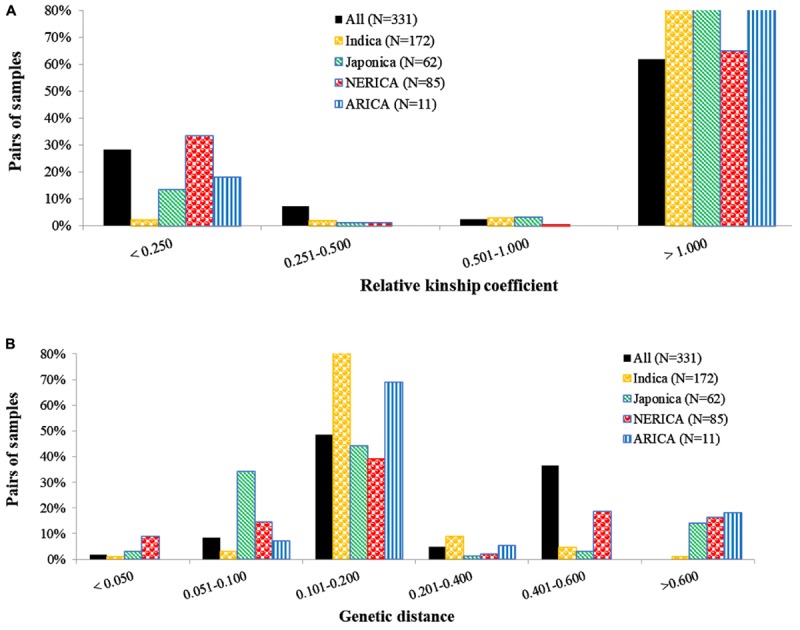
Distribution of pairwise **(A)** relative kinship and **(B)** identity-by-state based genetic distances among pairs of 330 rice genotypes, and within indica (172), japonica (62), NERICA (85), and ARICA (11) genotypes. The number of polymorphic SNPs (with minor allele frequency of ≥0.01) used for computing distance and kinship matrices were 15,020 SNPs for all 330 genotypes, 13,205 SNPs for indica, 11,808 SNPs for japonica, 13,601 SNPs for NERICA, and 10,275 SNPs for ARICA.

Kinship coefficients between pairs of genotypes varied from 0 to 1.96 (on a scale of 0–2), with an average of 1.05 (Supplementary Table S3). Overall, nearly 26 and 66% of the pairs of 330 genotypes had kinship values of ≤0.25 and >0.50, respectively. The proportion of highly related genotypes with kinship values greater than 0.50 varied across the four types of rice, with those belonging to the indica showing the highest (96.0%) and NERICAs with the lowest (65.4%) related pairs of genotypes (**Figure 1A**). Genetic distance between pairs of the 330 genotypes varied from 0.012 to 0.583, with an overall average of 0.277 (Supplementary Table S4). The 54,285 pairwise comparisons are summarized in **Figure 1B**, where most pairs had distances ranging from 0.1 to 0.2, but 941 pairs of genotypes (1.7%) had a genetic distance ≤ 0.05, and 88 pairs differed by ≤2% of the total number of alleles used in the analyses.

To identify the most similar pairs of genotypes within each group of rice, we computed genetic distance matrices among pairs of genotypes belonging to the indica, japonica, NERICA, and ARICA genotypes, which varied from 0.016 to 0.623, from 0.020 to 0.692, from 0.014 to 0.644, and from 0.075 to 0.763, respectively (Supplementary Table S4). Genetic distance for 9% of the pairs of 85 NERICAs was ≤0.05, a larger percentage of highly similar pairs compared with the 1.0% within pairs of indica, 3.1% within pairs of japonica, and none within pairs of ARICA (**Figure 1B** and Supplementary Table S4). The largest proportion of pairs of genotypes with genetic distance values >0.400 was observed within pairs of NERICAs (35.1% of the pairs) as compared with japonica (17.4% of the pairs), ARICAs (18.2% of the pair), and indica (5.6% of the pairs). Genetic distance between pairs of the 19 upland NERICAs varied from 0.017 to 0.215, with NERICA16 and NERICA18 and NERICA8 and NERICA9 differing by ∼2% of scored alleles. Genetic distance among pairs of 66 lowland NERICAs varied from 0.014 to 0.307, with 34 pairs of lowland NERICAs differing by ≤ 2% of alleles used for analyses (Supplementary Table S4).

**FIGURE 2:**
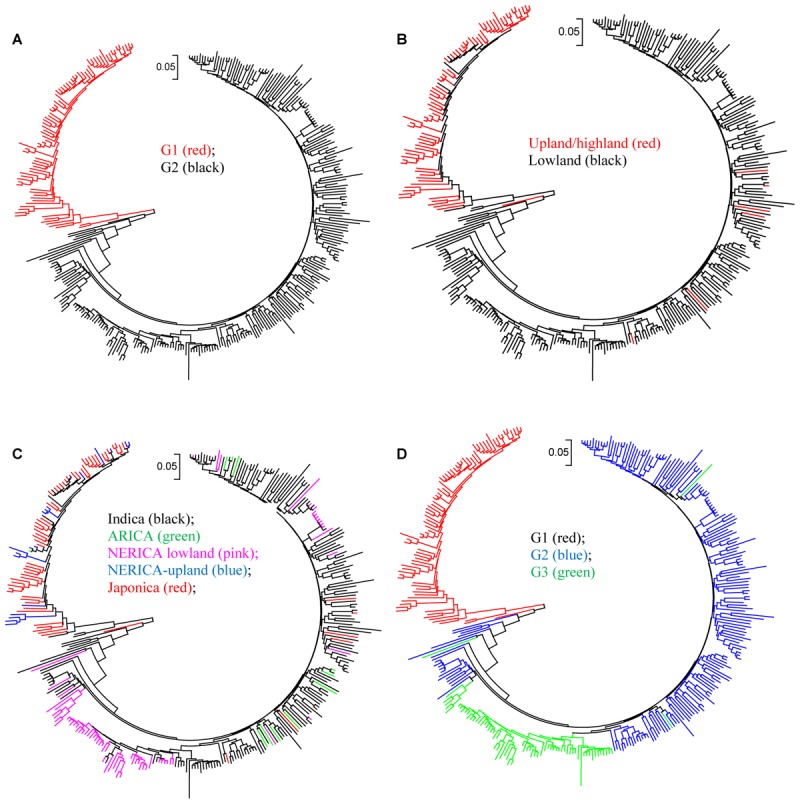
Neighbor joining tree for 330 rice genotypes based on identity-by-state genetic distance matrix computed from 15,020 SNPs: **(A)** groups based on cluster analysis; **(B)** groups based on ecology; **(C)** groups based on subspecies; and **(D)** groups based on STRUCTURE at *K* = 3. See Supplementary Table S1 and Supplementary Figure S2 for details.

**FIGURE 3:**
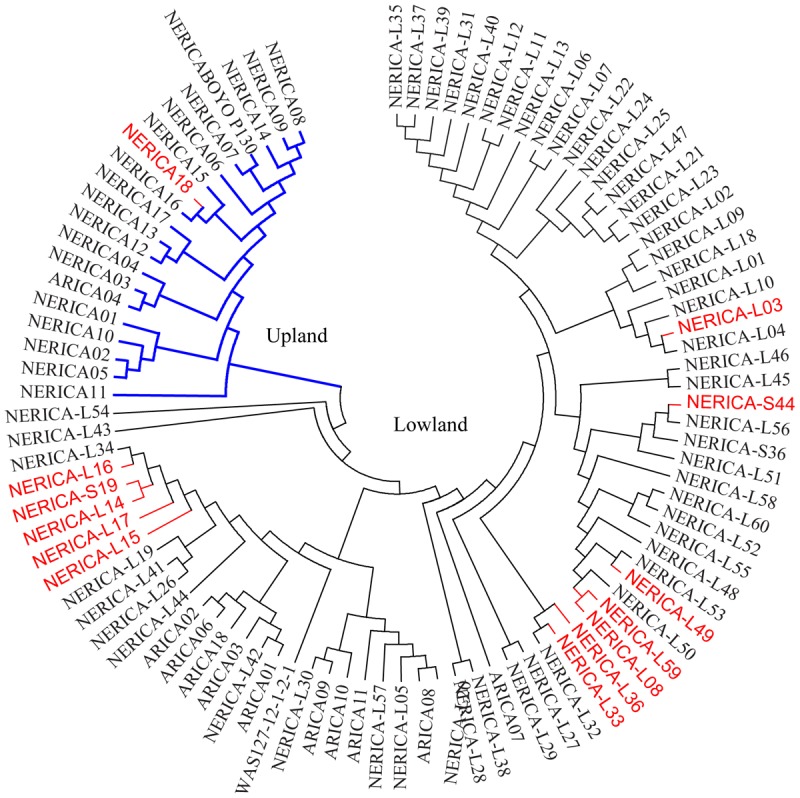
Neighbor-joining tree of 11 ARICA and 83 NERICA genotypes developed/released by Africa Rice Center based on genetic distance matrix computed from 13,849 SNPs that were polymorphic in these germplasm, each with minor allele frequency ≥0.010. Genotypes that showed ≥ 98% similarity with others are shown in red font.

The cluster analysis generated from the genetic distance matrix grouped the 330 genotypes into two major groups (**Figure 2A** and Supplementary Figure S2), which agrees with the upland and lowland ecologies (**Figure 2B**). The first group consisted of 81 genotypes that belong to upland (75) and lowland (6) ecologies. The second group had 7 upland and 243 lowland genotypes. Six genotypes from the upland and 7 genotypes from the lowland ecologies were mis-grouped based on the SNP data (**Figure 2B** and Supplementary Table S1). The indica, lowland ARICA and lowland NERICA genotypes were clearly separated from the japonica and upland NERICA and upland ARICA genotypes (**Figure 2C**). The cluster analysis also has shown the clear separation of the groups predicted based on the model-based population structure at *K* = 2 and *K* = 3 (**Figure 2D**), which is described in detail below. We observed groups of genotypes within indica, japonica, or NERICA that appeared to be highly similar. For each type of rice, we used the sphericity index curve in deciding the most informative number of genotypes to be retained and the redundant ones to be removed (Perrier et al., 2003). Using the sphericity index curve, 1 japonica, 11 indica, and 13 NERICAs (all marked in red in **Figure 3**) showed ≥98% similarity with one or more genotypes and were considered redundant (Supplementary Table S1). We then compared the genotypic data of the original 330 genotypes with the 304 non-redundant genotypes for polymorphism, allele frequency, genotype frequency, and genetic distance. Of the 15,020 SNPs that were polymorphic across the 330 genotypes, 99.8% of the markers (14,985 SNPs) were remained polymorphic within the 304 genotypes after filtering. Both allele and genotype frequencies were identical in both data sets (data not shown). The exclusion of 26 redundant genotypes reduced the number of pairs of genotypes that differed by <2% of the alleles from 88 (Supplementary Table S4) to just 22 (Supplementary Table S5).

### Population Structure and Genetic Differentiation

**FIGURE 4:**
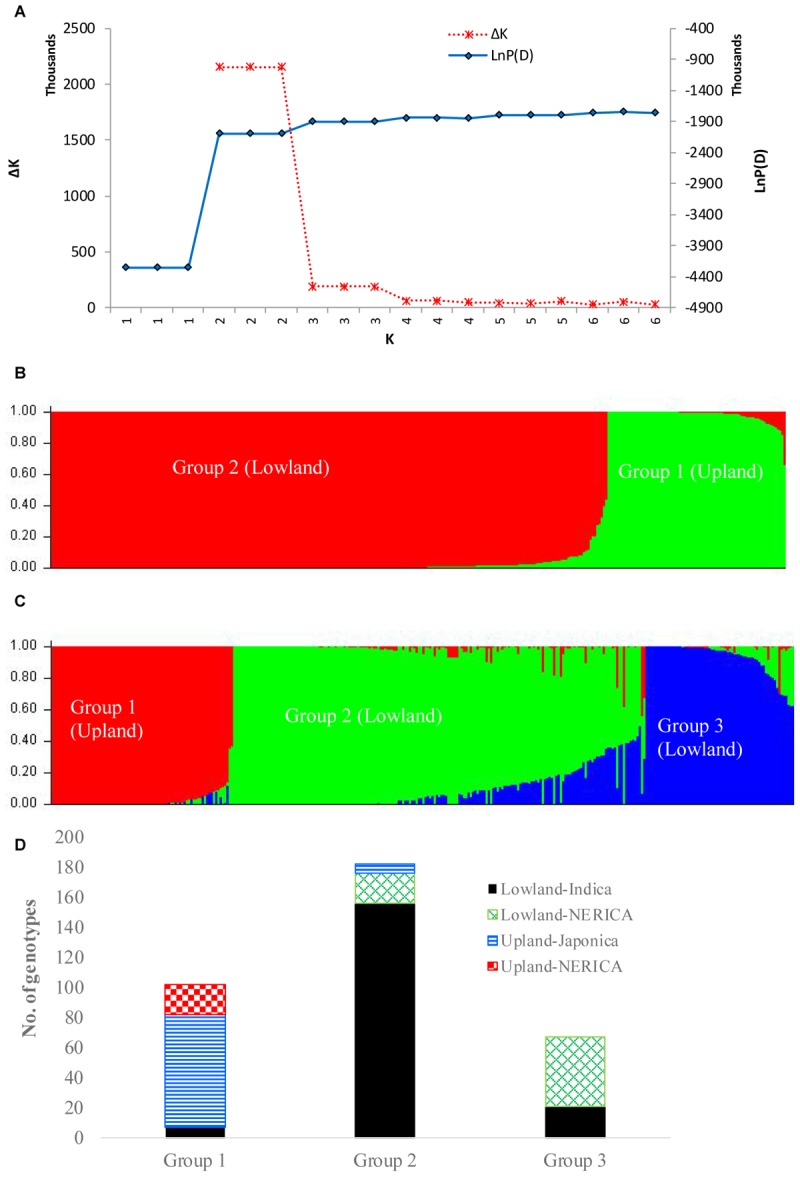
Population structure of 330 rice genotypes based on 15,020 SNP markers: **(A)** plot of LnP(D) and an *ad hoc* statistic Δ*K* calculated for *K* ranging from 1 to 6, with each *K* repeated thrice; **(B)** population structure at *K* = 2; **(C)** population structure at *K* = 3; and **(D)** number of lowland and upland NERICA, indica, and japonica genotypes that belong to each of the three groups predicted based on STRUCTURE at *K* = 3. In both **(B,C)**, each genotype is represented by a single vertical line that is partitioned into *K* colored segments in the *x*-axis, with lengths proportional to the estimated probability membership value (*y*-axis) at each of the *K* inferred clusters. See Supplementary Table S1 for group membership.

**FIGURE 5:**
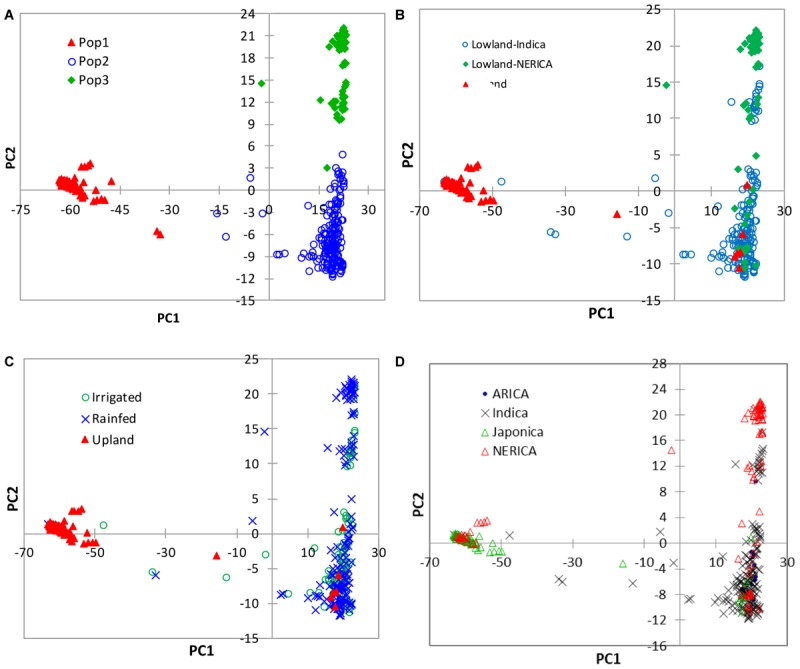
Plots of PC1 (56.7%) and PC2 (4.4%) from PCA analyses of 330 rice genotypes based on 15,020 SNPs. The plots were made using **(A)** group membership obtained from the model-based STRUCTURE at *K* = 3, **(B)** the lowland indica, lowland NERICA and upland ecologies, and **(C)** the irrigated lowland, rainfed lowland and upland ecologies, and **(D)** the different subspecies/types of rice. See Supplementary Table S1 for details.

In the model-based population structure analysis, the log probability of the data [LnP(D)] and *ad hoc* statistics Δ*K* both suggest the presence of two or three possible groups or sub-populations (**Figure 4A**) that was consistent with the rice growing ecologies (**Figure 4B** and Supplementary Table S1). At *K* = 2, a total of 77 genotypes belonging to the upland ecology form group 1, along with 5 lowland genotypes, while the second group consists of 245 genotypes adapted to lowland ecology along with 4 upland genotypes. At *K* = 3, the first group remained the same as the group membership obtained at *K* = 2. The first group consisted of 70% of japonica genotypes and 23% upland NERICAs derived from interspecific hybridization between *O. glaberrima* × *O. sativa*. The second group consisted of 178 lowland and 4 upland genotypes, of which 81% are indica and 11% are lowland NERICAs. The third group had 67 lowland genotypes of which 69% were interspecific lowland NERICAs and 30% indica (Supplementary Table S1). Results from PCA (**Figure 5**) demonstrated the presence of two distinct groups corresponding to the lowland and upland ecology, and a subgroup of the same genotypes forming the third STRUCTURE group obtained at *K* = 3 (Supplementary Table S1).

Single nucleotide polymorphisms diversity statistics indicate that NERICA genotypes were more diverse (higher polymorphism and nucleotide diversity) than ARICA, japonica, and indica genotypes (**Table 2**). When the analyses were done by dividing NERICAs into two ecologies, however, both polymorphism and nucleotide diversity in the lowland and upland NERICA genotypes were much lower than the indica and japonica genotypes. Groups predicted based on cluster and STRUCTURE analyses followed the pattern based on ecology of most genotypes in the group. Thus, genotypes belonging to the first cluster and STRUCTURE groups, which were predominantly upland japonica, showed lower polymorphism and nucleotide diversity than those genotypes in the second group, which were mostly lowland indica and NERICA. At *K* = 3, the third group, which primarily consisted of the lowland NERICAs, had the lowest polymorphism and nucleotide diversity as compared with the other two groups; however, this was probably due in part to a smaller sample size. Most of the molecular variation was observed between ecologies and groups predicted based on STRUCTURE and cluster analyses; very little was partitioned between rice types (**Table 3**).

**Table 2:** Summary of polymorphism and molecular diversity among 330genotypes that belongs to different categorical groups (types of rice, ecology, groups predicted based on the model-based STRUCTURE at *K* = 2 and *K* = 3, and cluster analysis).

Category	Description	Number of genotypes	Polymorphism with MAF ≥ 0.01 (%)	Average observed heterozygosity (Std)^∗^	Average expected heterozygosity (Std)^∗∗^	Average nucleotide diversity (Std)
Type of rice	Indica	172	87.9%	0.014 (0.064)	0.170 (0.143)	0.164 (0.078)
	Japonica	62	78.6%	0.015 (0.073)	0.224 (0.124)	0.186 (0.088)
	NERICA	85	90.6%	0.015 (0.065)	0.286 (0.144)	0.260 (0.123)
	ARICA	11	68.4%	0.023 (0.085)	0.255 (0.125)	0.174 (0.086)
Ecology	Irrigated lowland	88	88.3%	0.017 (0.067)	0.170 (0.144)	0.154 (0.073)
	Rainfed lowland	160	87.7%	0.013 (0.064)	0.147 (0.147)	0.144 (0.068)
	Upland	81	86.6%	0.014 (0.072)	0.175 (0.123)	0.154 (0.073)
	Lowland (Irrigated and rainfed)	248	92.0%	0.014 (0.064)	0.153 (0.143)	0.151 (0.071)
Groups based on ecology and type						
	Lowland Indica	183	89.1%	0.015 (0.065)	0.163 (0.145)	0.157 (0.074)
	Lowland NERICA	65	62.8%	0.020 (0.081)	0.149 (0.144)	0.105 (0.050)
	Upland NERICA	19	37.0%	0.031 (0.118)	0.263 (0.154)	0.097 (0.047)
	Upland Japonica	63	80.1%	0.015 (0.074)	0.202 (0.121)	0.165 (0.078)
Groups based on structure at *K* = 2 and cluster analysis						
	Group 1	82	73.0%	0.017 (0.079)	0.134 (0.136)	0.104 (0.049)
	Group 2	248	80.8%	0.015 (0.065)	0.143 (0.150)	0.135 (0.064)
Groups based on STRUCTURE at *K* = 3						
	Group 1	82	73.0%	0.017 (0.082)	0.138 (0.139)	0.098 (0.047)
	Group 2	181	72.7%	0.016 (0.068)	0.156 (0.152)	0.138 (0.065)
	Group 3	67	52.3%	0.021 (0.085)	0.120 (0.132)	0.079 (0.038)


**^∗^Std, standard deviation; ^∗∗^expected heterozygosity, gene diversity.**

**Table 3:** Analysis of molecular variance (AMOVA) for the extraction of molecular variation of 330 genotypes based on 15,020 polymorphic SNPs.

Category	Source of variation	d.f.	Sum of squares	Variance components	Percentage of variation
Groups based on STRUCTURE at *K* = 2 and cluster analysis	Among populations	1	742,206.2	3,007.2	76.0
	Within populations	658	626,670.4	952.4	24.0
	Total	659	1,368,876.6	3,959.6	100.0
Groups based on STRUCTURE at *K* = 3	Among populations	2	796,434.7	2,025.8	69.9
	Within populations	657	572,441.8	871.3	30.1
	Total	659	1,368,876.5	2,897.1	100.0
Lowland and upland ecologies	Among populations	1	621,554.3	2,516.9	68.9
	Within populations	658	747,322.3	1,135.7	31.1
	Total	659	1,368,876.6	3,652.6	100.0
Indica lowland, NERICA lowland, and upland	Among populations	2	649,553.4	1,657.0	60.2
	Within populations	657	719,323.2	1,094.9	39.8
	Total	659	1,368,876.6	2,751.9	100.0
Indica lowland, NERICA lowland, NERICA upland, and Japonica upland	Among populations	3	65,534.9	1,609.3	59.7
	Within populations	656	713,534.7	1,087.7	40.3
	Total	659	779,069.6	2,697.0	100.0
Irrigated lowland, rainfed lowland, and upland ecologies	Among populations	2	629,557.5	1,512.5	57.6
	Within populations	651	725,858.2	1,114.9	42.4
	Total	653	1,355,415.7	2,627.4	100.0
Indica, Japonica, NERICA, and ARICA	Among populations	3	422,336.3	1,011.8	41.2
	Within populations	656	946,540.3	1,442.9	58.8
	Total	659	1,368,876.6	2,454.7	100.0
NERICA and ARICA	Among populations	1	7,747.8	151.3	7.5
	Within populations	188	350,241.7	1,862.9	92.5
	Total	189	357,989.5	2,014.2	100.0


**FIGURE 6:**
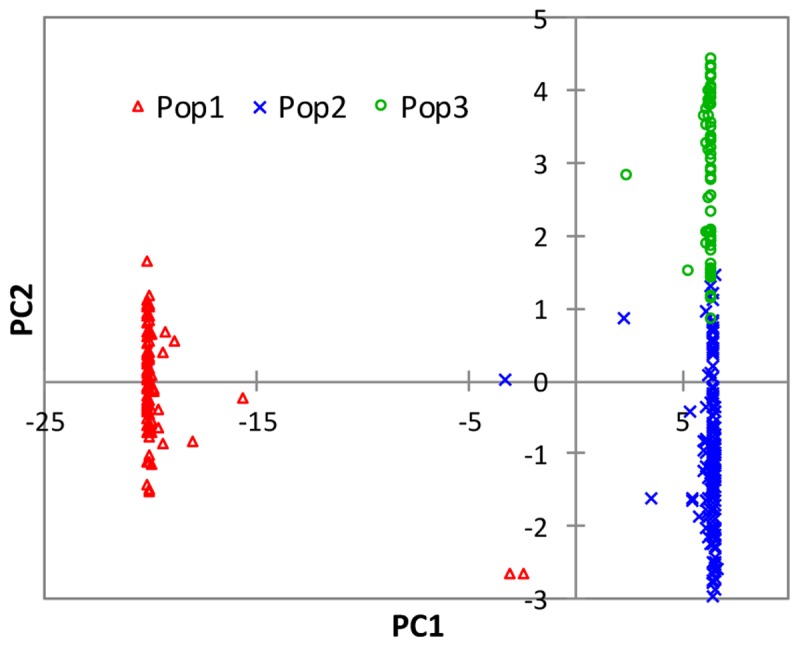
Plots of PC1 (73.1%) and PC2 (1.9%) from PCA analyses of 330 rice genotypes based on 1,197 SNPs that showed significantly different (*p* < 0.05) *F*_ST_ values between the lowland and upland ecologies. See Supplementary Table S1 for details.

The partitioning of the overall molecular variance into different hierarchical levels revealed that differences in types of rice, ecologies, and groups predicted based on cluster analysis and the model-based population structure accounted for 41.2%, 57.6–68.9%, and 69.9–76.0% of the total variation, respectively (**Table 3**). A permutation tests indicated that the proportion of variance attributable for all hierarchical levels were highly significant (*p* < 0.001). To investigate the extent of genetic differentiation among hierarchical levels, we compared *F*_ST_ values between pairs of the different types of rice, ecologies, and predicted groups based on both cluster and the model-based STRUCTURE analyses (Supplementary Table S6). *F*_ST_ values were highly variable among the predicted groups based on STRUCTURE at *K* = 3, with group 1 (upland ecology) showing the highest divergence as compared with both group 2 (0.759) and group 3 (0.834); groups 2 and 3 showed less divergence (*F*_ST_ 0.227). We found little differentiation between irrigated and rainfed lowland ecologies (0.015), and between indica and ARICA (0.030); moderate differentiation between NERICA and ARICA (0.075), and between indica and NERICA (0.112). There was very great genetic differentiation between indica and japonica (0.624), between japonica and NERICA (0.449), between japonica and ARICA (0.588), between the lowland and upland ecologies (0.688). In all multivariate analyses, we observed clear population structure between the lowland (irrigated and rainfed lowland) and upland ecologies. To identify SNP markers that contributed to the major genetic differentiation between the lowland and upland ecologies, we used the hierarchical island model to identify loci that may have undergone selection. We found out that approximately 8% of the SNPs (1,197 of the 15,020 SNPs) were significantly (*p* < 0.05) different between the lowland and upland ecologies (Supplementary Table S7). The 1,197 SNPs were sufficient to clearly separate the three groups (**Figure 6**) in the same way as predicted based on the model-based STRUCTURE at *K* = 3 (**Figure 4**).

## Discussion

### Genetic Purity

Rice is a selfing species with an outcrossing rates ranging from 2 to 5% (Semon et al., 2005). As a result, most genotypes used in the current study were expected to display less than the average residual heterozygosity expected in S_5_ generation, which was seen with ∼95% of the genotypes. Only about 4% (13 of 330 genotypes) had residual heterozygosity exceeding 5%, which is higher than expected in the absence of human error, which includes ARICA18 (5.9%), NERICA-L38 (7.4%), and NERICA-L54 (16.3%), eight indica genotypes, and two japonica genotypes (Supplementary Table S1). Residual heterozygosity is common in many rice varieties and has been reported to be useful as sources of genetic variation in parental lines used in developing new segregating populations (Belefant-Miller et al., 2012). However, the availability of genetically pure seeds is one of the important quality control criteria in breeding and seed production, which directly affects grain quality for commercialization (Semagn et al., 2012; Ertiro et al., 2015). Even a small proportion of contamination in seed sources could lead to variation in agronomic performance, reducing the quality of the product for marketing. Rouging off-types and voluntary plants in seed production plots minimizes such issues but incurs additional effort and cost. Maintenance of genetic purity of parental seed sources by reducing residual heterozygosity to less than 5% is an efficient and cost-effective method to maintain quality seed production. Genotypes with residual heterozygosity >5% should be purified if they will be used as trait donors and genome-wide association studies.

### Genetic Relatedness and Relationships

Kinship values estimated between pairs of genotypes using either known pedigree relationships or genome-wide molecular markers are useful to understand the extent of relatedness (Eu-ahsunthornwattana et al., 2014). Kinship values close to zero indicate unrelated germplasm, while those close to 0.5 or higher (which around 66% of the pairs of genotypes in this study) refer to full sibs or highly similar germplasm (Dodds et al., 2015). Groups of closely related parents tend to bring redundant genetic value to a breeding program, which is evident in NERICAs due to the repeated use of four *O. glaberrima* and eight *O. sativa* parents in developing 66 lowland, and 19 upland NERICAs. In fact, some of the parents, such as TOG5681 and IR64 were extensively used in 63 and 71 crosses, respectively (Supplementary Table S1).

We observed highly variable genetic distances among pairs of genotypes belonging to the ARICA, NERICA, indica, and japonica groups. None of the ARICAs used in the present study shared the same pedigree, which is clearly evident from the huge genetic difference (range 0.075–0.763) observed among pairs of these varieties. The 19 upland NERICAs were derived from interspecific crosses involving a common *O. glaberrima* CG14 and three *O. sativa* (WAB56-50, WAB56-104, and WAB181) parents (Supplementary Table S1). Highly similar NERICA8 vs. NERICA9 and NERICA16 vs. NERICA18 are pairs of sister lines extracted from WAB56-104/CG14//2^∗^WAB56-104 and CG14/WAB181-18//2^∗^WAB181-18, respectively. Using 102 microsatellite markers on 18 upland NERICAs, we previously reported wider genetic variation among most pairs of upland NERICAs except NERICA8 and NERICA9 that were found to be nearly identical at the molecular level (Semagn et al., 2006). The 66 lowland NERICAs were developed from interspecific crosses involving three *O. glaberrima* (TOG 5681, TOG 5674, and TOG 5675) and five *O. sativa* (IR64, IR28, IR31785-58, IR1529-680, and IR31851-96) parents. TOG 5681 and IR64 were used as parents in 63 and 60 of the lowland NERICAs, respectively (Supplementary Table S1), which resulted in multiple sister lowland NERICAs with a common pedigree, the same level of inbreeding, and the same adaptation. Twelve of the 66 lowland NERICAs (NERICA-S44, NERICA-L17, NERICA-L14, NERICA-L15, NERICA-L36, NERICA-L03, NERICA-L33, NERICA-L16, NERICA-L59, NERICA-S-19, NERICA-L08, and NERICA-L49), differ from at least one other NERICA by <2% of scored alleles, and have been considered redundant based on the maximum length subtree method (Supplementary Table S1). Despite the common parentages, these lines exhibited large phenotypic variability, which formed the basis for their releases as different varieties. Thus, these lines are evidently different for some key genes, and may even be used as nearly isogenic pairs to determine genomic regions causing this phenotypic diversity, once the differences are confirmed in further phenotypic studies.

### Population Structure and Genetic Divergence

Several studies reported clear population structure in Asian rice based on subspecies/ecotypes (Glaszmann, 1987; Zhang et al., 1992; Garris et al., 2005; Zhao et al., 2011). Our results from cluster analysis (**Figure 2**), the model-based population structure (**Figure 4**), and PCA (**Figure 5**) revealed clear population stratification consistent with ecological adaptation and ecotypes. Cluster analysis clearly separated the upland genotypes from the lowland genotypes. All japonica genotypes in our study are upland types, and most (75%) clustered together with all 19 upland NERICAs derived from three japonica parents. The 66 lowland NERICAs derived from five indica parents clustered together with the intraspecific lowland ARICA and lowland indica genotypes. The model-based structure at *K* = 3 and PCA further divided the lowland genotypes into two subgroups corresponding indica (group 2) and most interspecific lowland NERICA (group 3) genotypes. This is likely because while the NERICA are predominantly *O. sativa* (indica) background following backcrossing, they contain a low proportion of *O. glaberrima* alleles from the initial cross, which was tracked in a previous study using 60 microsatellite markers (Ndjiondjop et al., 2008). Depending on the number of backcrosses used, the average remaining *O. glaberrima* genome contribution varied from 7.2 to 8.5%. The lowland NERICAs with the smallest introgression from the *O. glaberrima* parent were the most similar to the lowland indica compared to their interspecific sister lines.

The three main rice ecologies across west and central Africa are upland, the rainfed lowlands, and the irrigated lowlands (Maclean et al., 2002) which, respectively, account for ∼44, 31, and 12% of the total rice production area in the region. These ecologies differ significantly in terms of the severity of drought, soil fertility, soil acidity, iron toxicity, diseases and pests, and desirable agronomic traits (i.e., maturity, plant height, and yield potential). Upland rice is grown in well-drained soils with variable topographies ranging from sloping lands with high runoff to low-lying valleys and flat lands. These areas are characterized by poor soil physical and chemical properties, including low soil fertility and high soil acidity, and erratic precipitation. Most upland rice varieties are thus characterized by early maturity, deep roots system, and a higher tolerance to drought and acidic soils; however, they have low yield potential (on average about 1 t ha^-1^) and tend to lodge under high-levels of fertilizer and supplemental irrigation.

Rainfed lowland systems are more robust than upland systems, with good potential for intensification. Rice yields in rainfed lowlands are substantially higher than those in the rainfed uplands, but nevertheless still on average about 2 t ha^-1^; however, there is a possibility of higher yield potential for these genotypes with increased external inputs, including supplemental irrigation. Most cultivars grown in the irrigated lowland ecology have a short stature to avoid lodging under high fertilizer input and can produce over 5 t ha^-1^. Although rice genotypes have clear differences in morphological, phenological and other traits for adaptation to upland, irrigated lowland and rainfed lowland ecologies, we only observed moderate genetic differentiation (*F*_ST_ = 0.015) between the rainfed lowland and irrigated lowland ecologies as compared with the very great genetic differentiation between irrigated and upland (0.680) and rainfed and upland (0.70) ecologies (Supplementary Table S6).

Indica genotypes are predominantly adapted to the lowland tropical and subtropical regions, while japonica rice is more adapted to the upland and highland temperate and tropical regions. In the present study, we observed similar levels of polymorphism and diversity within indica and japonica (**Table 2**), which is in some disagreement with other studies (Zhang et al., 1992; Garris et al., 2005) who reported higher genetic variation and haplotypes within the indica than japonica groups. The present study found 710 SNPs with *F*_ST_ values ranging from 0.904 and 0.941 and were sufficient to clearly differentiate the indica and japonica genotypes (except nine genotypes). Previous studies were also able to assign most rice lines into their respective subspecies groups using molecular markers (Morishima and Oka, 1981; Glaszmann, 1987; Zhang et al., 1992). The high genetic differentiation between the indica and japonica, and also between upland and lowland ecologies may also be partly due to the autogamous breeding system, which plays a significant role in structuring the genetic variation within and among hierarchical groups or populations. Generally, outcrossing promotes gene flow, which results to higher polymorphism and greater genetic diversity, while selfing restricts gene flow and leads to more genetic differentiation among populations (Hamrick and Godt, 1997). The large amount of genetic differentiation attributable to differences among groups (ranging from 41.1 to 76.0%) observed in the present study (**Table 3**) agrees with previous studies.

## Conclusion

This is the first study that characterized the molecular variation, relatedness and population structure of NERICA and ARICA varieties developed by the AfricaRice in comparison with various indica and japonica varieties widely used in sub Saharan Africa. Overall, fourteen out of the 96 NERICAs and ARICAs (NERICA18 from the upland ecology and 13 of the 66 lowland NERICAs) differ from at least one other NERICA by <2% of scored alleles, which is an indication of their narrow genetic difference from other sister genotypes. Thus, these lines are highly similar at the molecular level, but were released as phenotypically different varieties that may be due to differences for some key genes, and may even be used as nearly isogenic pairs to determine genomic regions causing this phenotypic diversity, once their phenotypic variation is determined in future through multilocation phenotyping under the same management conditions. Results from this study (1) suggest the need in diversifying parental lines for new breeding programs to develop genetically diverse varieties, such as NERICA-11 from upland ecology, NERICA-L43 and NERICA-L54 from lowland ecology; (2) could aid breeders in selecting the most genetically divergent varieties as the best parental combinations for new breeding programs, provided the selected genotypes also have the desired phenotypic traits of interest.

## Data Availability

All relevant files are included within this article and its additional files.

## Author Contributions

MNN conceived, designed, and supervised the experiments, secured funding, and partly drafted the paper. AG, SK, EP, and PW were responsible for the sample preparation, DNA extraction, and/or compilation of passport information. KS analyzed the data and wrote most part of the paper. MSo, MSi, and BM provided valuable suggestions on the paper. MW contributed to and edited the paper. All authors read and approved the paper.

## Conflict of Interest Statement

The authors declare that the research was conducted in the absence of any commercial or financial relationships that could be construed as a potential conflict of interest.

## Footnotes

1 http://www.africarice.org/warda/ARICA.asp

2 http://www.diversityarrays.com/submission-sample

3 www.xlstat.com
