# Supplementary material for: Assessment of Genetic Variation and Population Structure of Diverse Rice Genotypes Adapted to Lowland and Upland Ecologies in Africa Using SNPs
Source: Front Plant Sci. 2018 Apr 9;9:446. doi: 10.3389/fpls.2018.00446 (PMC5900792; doi:10.3389/fpls.2018.00446)
Supplement: Supplementary file 8 [file Image_1.PDF]

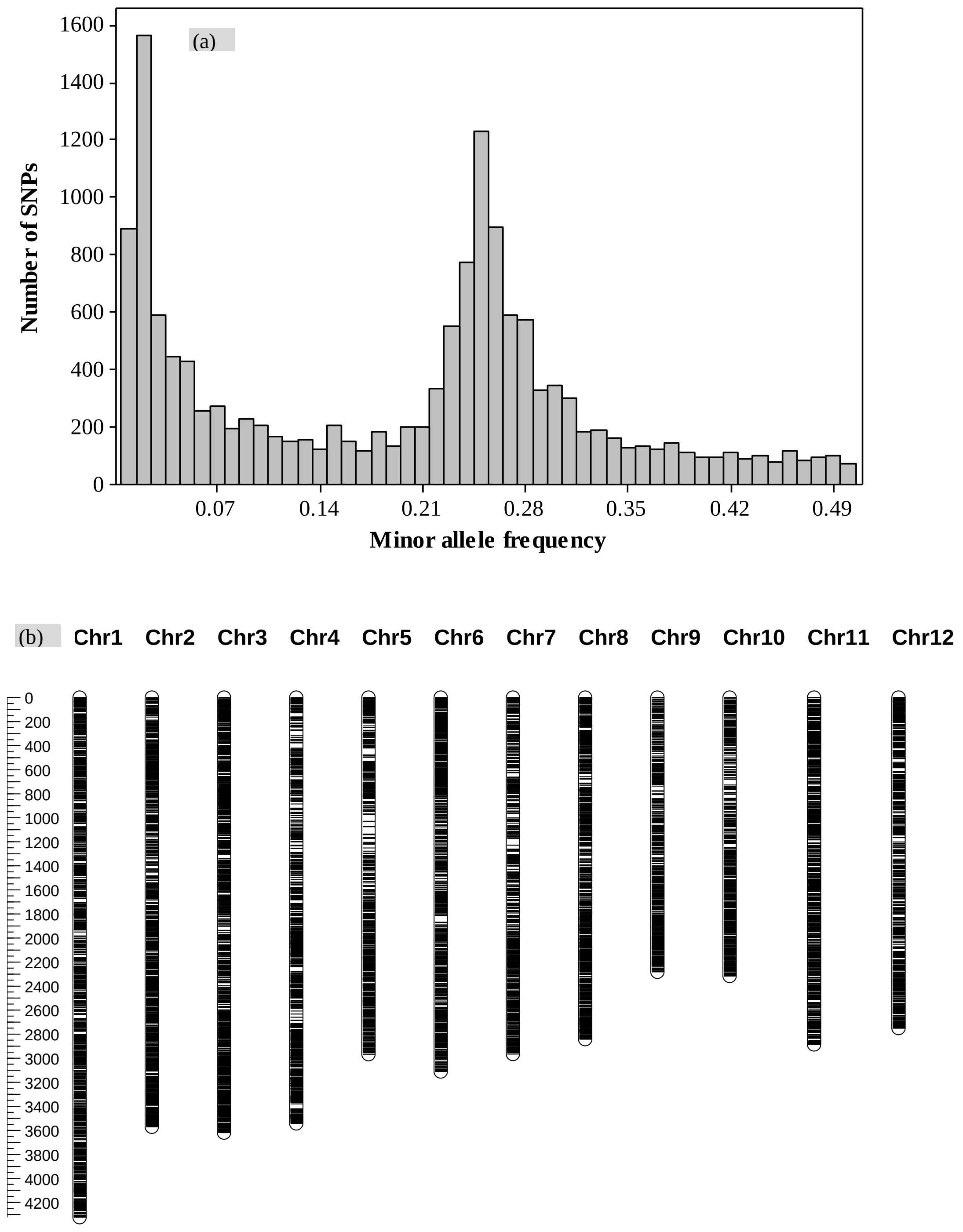

Supplementary Figure S1 (a) summary of minor allele frequency distribution for the 15,020SNPs that were polymorphic across 330 rice genotypes, and (b) their chromosomal distribution and physical map positions. Map position (x 10,000 bp) is shown on the left side. For each chromosome, the horizontal line represents a single SNP; the black shaded regions indicate high marker density due to presence of multiple SNPs with small physical distance, while the white regions indicate lower marker density in that interval.
