# Supplementary material for: Assessment of Genetic Variation and Population Structure of Diverse Rice Genotypes Adapted to Lowland and Upland Ecologies in Africa Using SNPs
Source: Front Plant Sci. 2018 Apr 9;9:446. doi: 10.3389/fpls.2018.00446 (PMC5900792; doi:10.3389/fpls.2018.00446)
Supplement: Supplementary file 9 [file Image_2.PDF]

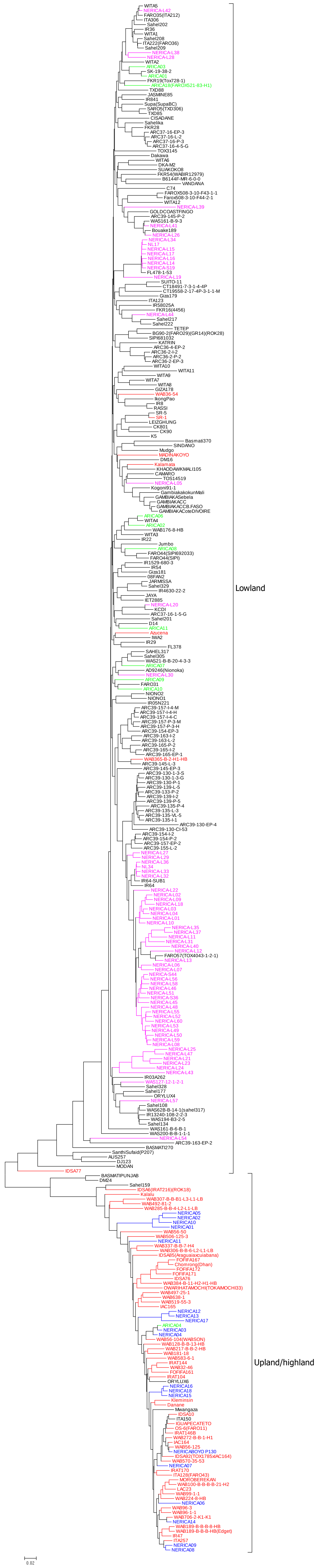

Supplementary Figure S2 Neighbor-joining tree of 330 genotypes based on identity by state based genetic distance matrix computed from 15,020 polymorphic SNPs. Genotypes that belong to the same group are shown by the same color: indica (black font), japonica (red), ARICA (green), NERICA lowland (pink), and NERICA upland (blue) genotypes. See Supplementary Table S1 for details.
